# Diagnosis and Management of Febrile Neutropenia in Pediatric Oncology Patients—A Systematic Review

**DOI:** 10.3390/diagnostics12081800

**Published:** 2022-07-25

**Authors:** Estera Boeriu, Alexandra Borda, Dan Dumitru Vulcanescu, Vlad Sarbu, Smaranda Teodora Arghirescu, Ovidiu Ciorica, Felix Bratosin, Iosif Marincu, Florin George Horhat

**Affiliations:** 1 Department of Pediatrics, “Victor Babes” University of Medicine and Pharmacy, Eftimie Murgu Square 2, 300041 Timisoara, Romania; estera.boeriu@umft.ro; 2 Department of Oncology and Haematology, “Louis Turcanu” Emergency Clinical Hospital for Children, Iosif Nemoianu Street 2, 300011 Timisoara, Romania; borda.alexandra@gmail.com; 3 Multidisciplinary Research Center on Antimicrobial Resistance (MULTI-REZ), Microbiology Department, “Victor Babes” University of Medicine and Pharmacy, 300041 Timisoara, Romania; dannvulcanescu@gmail.com (D.D.V.); sa.vlad1503@gmail.com (V.S.); horhat.florin@umft.ro (F.G.H.); 4 Business Administration and Economics Faculty, West University of Timisoara, Johann Heinrich Pestalozzi Street 16, 300115 Timisoara, Romania; 5 Methodological and Infectious Diseases Research Center, Department of Infectious Diseases, “Victor Babes” University of Medicine and Pharmacy, 300041 Timisoara, Romania; felix.bratosin7@gmail.com (F.B.); imarincu@umft.ro (I.M.)

**Keywords:** febrile neutropenia, pediatric oncology, broad-spectrum antibiotics

## Abstract

Infectious diseases are associated with a high morbidity and mortality rate among pediatric cancer patients undergoing treatment or receiving a transplant. Neutropenia represents a potentially fatal complication of cancer treatment and is associated with a high risk of developing bacterial infections. Although febrile neutropenia (FN) can affect both adults and children, the latter has a higher chance of infections with an unknown origin. Prompt empiric broad-spectrum antibiotic administration is collectively considered the best therapeutic approach. This review aims to analyze the latest works from the literature regarding the therapeutic strategies, schemes, and approaches and the efficacy of these in pediatric febrile neutropenia. Following PRISMA guidelines, an advanced search on PubMed, Scopus, and Cochrane Library, using the keywords “febrile neutropenia”, “pediatric”, “cancer”, and “oncology”, was performed. A total of 197 articles were found to be eligible. After screening the abstracts and excluding unfit studies, 16 articles were analyzed. There were eight retrospective studies, five prospective studies, and two clinical trials. Altogether, these studies have described around 5000 episodes of FN. The median age of the participants was 7.6 years, and the underlying condition for most of them was acute leukemia. The infectious agent could only be determined in around one-fifth of cases, from which 90% were of bacterial origin. As such, empirical broad-spectrum antibiotics are used, with the most used treatment scheme comprising third- and fourth-generation cephalosporins and antipseudomonal penicillins. In order to improve the treatment strategies of FN episodes and to successfully de-escalate treatments toward narrower-spectrum antibiotics, hospitals and clinics should increase their efforts in identifying the underlying cause of FN episodes through blood culture urine culture and viral tests, wherever infrastructure enables it.

## 1. Introduction

Infectious diseases are associated with a high mortality and morbidity rate among pediatric cancer patients undergoing treatment for their oncological condition or among patients that have received a stem cell transplant [1,2,3]. Neutropenia, defined as the absolute neutrophil count (ANC) below 500 cells/mm^3^ or ANC expected to drop below 500 cells/mm^3^ in the next 48 h, represents a potentially fatal complication of cancer treatment and is associated with a high risk of developing bacterial infections. Fever is defined as having a bodily temperature above 38.3 °C, measured orally, or two measurements above 38 °C that are taken at different time points, at least an hour apart [4,5,6]. While febrile neutropenia is a condition affecting both adult and pediatric patients, there are several differences between the two age groups. An important one is that children have higher chances of developing bacterial infections with an unpinnable source [5,7,8]. The recommended empirical treatment is dependent on the risk group to which the patients have been assigned. Febrile neutropenic patients are considered to be high risk if they exhibit one or more of the following risk factors: CRP ≥ 90 mg/L, hypotension, relapsed leukemia, platelet count below 50,000 cells/mm^3^, or the elapsed time between the end of chemotherapy and the beginning of fever being less than seven days [1,4,9]. Patients not meeting any of the aforementioned criteria are included in the low-risk group, while those meeting criteria and having sustained fever and neutropenia for over 96 h are defined as having persistent high-risk febrile neutropenia [6,10]. Either way, prompt empiric broad-spectrum antibiotic administration is collectively recommended by the FN guidelines and professional medical societies [11,12,13]. The increased duration until antibiotic administration is initiated has been associated, in the pediatric setting, with higher rates of sepsis and higher rates of intensive care admission and death, thus prompting the term “golden hour” as the recommended timeframe between FN patient presentation to the start of the antibiotic administration [7,14].

This study aimed to analyze the latest works in the literature regarding the therapeutic strategies, treatment schemes, and approaches in pediatric febrile neutropenia.

## 2. Materials and Methods

### 2.1. Study Design

The current systematic review was included in the PROSPERO registry for systematic review protocols [12] and followed the Preferred Reporting Items for Systematic reviews and Meta-Analyses (PRISMA) guidelines [15] for providing a comprehensive overview of the management of febrile neutropenia in pediatric oncologic patients. An advanced search was conducted on PubMed, Scopus, and Cochrane Library, using the keywords “febrile neutropenia”, “pediatric”, “cancer”, and “oncology”. We reviewed data from the literature, presented as reviews and original articles, covering the period from 1 May 2018 to 1 May 2021, resulting in 197 eligible articles. After reading the abstracts, 170 studies were excluded. Thirty-nine duplicates were removed by using EndNote. Only papers in English or Romanian were included after further reading of the remaining studies, resulting in another 11 papers being excluded. In the end, 16 studies were chosen to be analyzed.

This review of the literature aimed to answer the following questions:

- Which is the optimal treatment strategy in cases of febrile neutropenia?
- Which type of bacteria are most often involved in febrile neutropenia?

### 2.2. Selection Criteria

Papers retrieved during the searches were checked against the following inclusion criteria: (1) full-text original work published in a peer-reviewed journal; (2) articles featuring only oncologic pediatric patients; (3) articles featuring microbiological profiling; (4) articles featuring antimicrobial therapies for FN patients; and (5) articles written in English or Romanian.

### 2.3. Data Extraction

Two investigators (D.D.V. and V.S.) independently screened each title and abstract in accordance with our inclusion and exclusion criteria. Any difference between the two investigators in the screening process was resolved by either discussion or a third senior investigator (E.B.). If doubt persisted, the article was included in the full read set. The following data items were collected from the articles: the number of FN episodes, patient characteristics (age and gender), type of malignancy, ANC levels before antibiotic treatment, observed infection locations, etiological agents, and treatment plans (antimicrobial, antiviral, and antifungal) and outcomes (successful treatment, deaths, ICU admissions, sepsis cases). All data were extracted from article texts, tables, figures, and online

### 2.4. Quality Assessment

Two investigators (A.B. and F.B.) independently assessed data from papers and documented findings by using the Study Quality Assessment Tools published by the NHLBI. The tools are specific to study designs and test for potential flaws in study methods or implementation. The Quality Assessment of Controlled Intervention Studies tool was used for randomized controlled studies (RCT), while the Quality Assessment Tool for Observational Cohort and Cross-Sectional Studies was used for the rest of the studies [10]. For each of the 14 questions in a tool, “Yes” answers counted as 1 point, while a “No” or “Other” answer counted as 0 points. Then the final quality score was summed up. As such, studies having a grade between 0 and 4 were considered to be of poor quality, studies with a grade between 5 and 9 were considered to be of fair quality, and studies of 10 and above were considered to be of high quality.

## 3. Results

### 3.1. Overview

**Figure 1:**
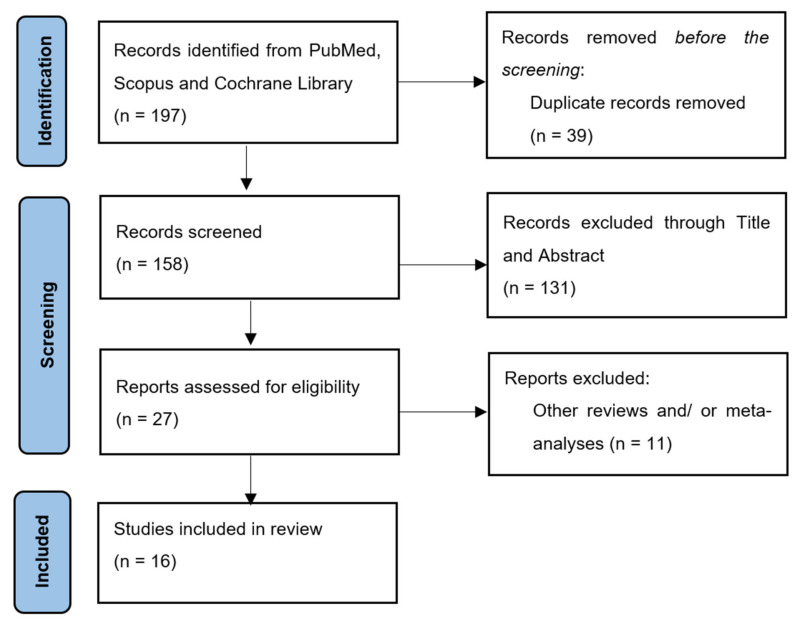
PRISMA flowchart for the selection process.

This study analyzed the results of 16 different studies, half of which were retrospective, one was a review, two were clinical trials, and the rest were prospective studies. The flow diagram is presented in Figure 1, while the data of the 16 studies included are presented in Table 1.

For the studied period, 197 total records were found when searching on PubMed, Scopus, and Cochrane Library. Thirty-nine of these records were observed to be duplicates across the studied databases, which resulted in 158 studies, whose title and abstract were screened, as previously described in Section 2.3. This led to 131 total records being dismissed. Most (*n* = 119) were excluded, as they did not study febrile neutropenia in the pediatric population with oncological conditions. The other 22 were dismissed, as they featured other forms of neutropenia. This resulted in 27 records being fully analyzed. Out of those, two were excluded for being letters to the editor, while nine were excluded due to our language restrictions, resulting in sixteen final articles.

Altogether, these studies have described around 5000 (*n* = 4958) episodes of FN in more than 2500 patients (*n* = 2664), resulting in an average of 1.86 episodes per patient. In regard to quality assessment, most studies were considered of high quality, scoring more than 10 out of 14 points on our quality assessment tools. The median score across all studies was 11. Demographic data and the main outcomes of each study can be seen in Table 2. Male patients were more common, accounting for 55.74% of the total cases. The median age of the participants in these studies was 7.6 years, and the underlying condition for most of them was acute leukemia (30.71%), either lymphoblastic (22.72%) or myeloid (7.99%).

Although most studied patients had an ANC lower than 500 (94.79%), only studies 1, 3, 4, 5, 9, 10, 11, and 14 presented a mean value for their studied patients, which was 161.5 on average. Regarding sepsis, eight studies did not report any such incident; however, in studies where this situation was encountered, sepsis cases sum up to 85 episodes (4.91%). ICU admission was not reported in five records, while where it was reported, it accounted for 252 episodes (10.43%). Regarding outcome, 3561 (71.82%) out of the 4958 total episodes were treated, while 89 (3.34%) out of the 2664 total patients had died.

The infectious pathogen was determined in only about one-fifth of the cases, while in the rest of roughly 80% of the described episodes, the cause of infection could not be singled out. In the instances where the cause of infection was established, a bacterium was involved around 90% of the time. 

When it comes to the documented cases of infection, most types encountered were BSIs in 914 episodes (18.43%), followed by localization in the chest (*n* = 144 episodes, 15.33%). Six studies did not report such data. The median hospital stay was reported in nine papers and was around one week, mostly ranging from 4.75 days to 20 days. Bacterial infection was observed in 928 episodes, fungal infection in 95 episodes, and viral infection in 41 episodes; however, four studies did not report any microbiological profiling. Moreover, seven studies reported on MDR strains, which summed up 159 episodes (17.13% of bacterial infections). Our main findings in relation to etiology are recorded in Table 3.

Regarding the main antibiotics used in the empirical treatment of FN episodes, most schemes comprise cephalosporins (third and fourth generation) and antipseudomonal penicillins, namely Piperacillin in combination with Tazobactam, a β-lactamase inhibitor to which, in certain schemes, aminoglycosides are added in order to expand the covered spectrum. Few of the analyzed studies strayed from this path and administered carbapenem-type antibiotics. 

The most used antibiotic was Cefepime, as it appeared as the first treatment option in five studies and as the second treatment option in three. While Ceftazidime was used more as first option (three studies), Piperacillin/Tazobactam was used more as a second option treatment (four studies). Other important antibiotic drugs used were Vancomycin and Amikacin, as they usually were paired with another antibiotic drug. A few studies (nos. 1, 5, 7, 10, and 12) also reported the average time to antibiotic administration, which is crucial when it comes to bacterial infections in these patients. In most studies, except for number 12, drug administration was performed in 120 min or less. Other anti-infectious medications were represented by both antivirals, but more so by antifungals. The main findings in regard to treatment options are presented in Table 4

The main outcomes extracted from the studied papers are presented in Table 5. A few studies (nos. 3, 4, 11, and 15) described cases of persistent fever even after finalization of treatment, totaling 15 cases. Record numbers 7, 13, and 16 also had patients who required removal of their central line, which summed up to 62 patients. 

### 3.2. Definition of Febrile Neutropenia

The definitions of what is considered a fever and neutropenia, respectively, vary slightly in the medical community. It is generally acknowledged, though, that an ANC < 500 cells/mm^3^ means neutropenia, but ANC up to 1000 cells/mm^3^ is accepted if that number is expected to drop to <500 cells/mm^3^ in a few days (usually 48 to 72 h). The value by which fever is defined varies somewhat more and is divided into two groups: single body temperature measurement and seriated measurements. 

Accepted values for single measurements are between >38.3 °C and >38.5 °C, but certain studies [4] consider values as low as ≥37.8 °C in their definitions of fever. Multiple readings >/≥38 °C at least one hour after the first reading are also considered to be fever; one study [15] considered three temperature measurements between 37.5 and 38 °C over 24 h to be fever [2,4,15,16,17,18,19,20,21,22,23,24,25].

### 3.3. Microbiological Profile of FN

**Figure 2:**
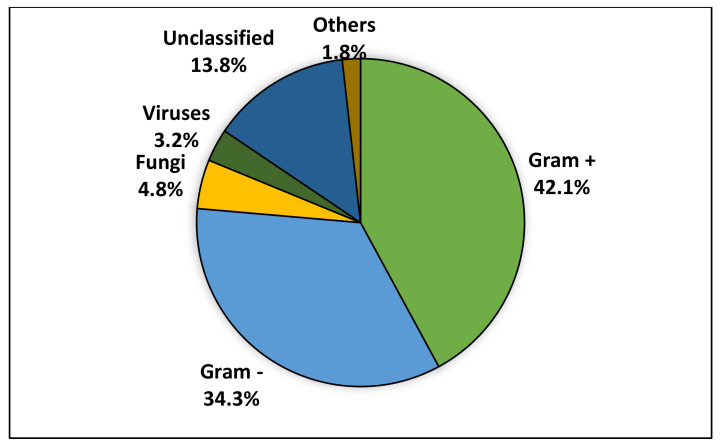
Pathogens involved in the microbiologically documented infections.

As aforementioned, in the large majority of the studied cases, the underlying pathogen could not be established, and in the few cases where a microorganism was identified, it was usually a bacterium. One study [18] that analyzed 563 episodes of FN confirmed that, in 32 (5.68%) of them, the pathogenic organism turned out to be a virus. Taken as a whole, out of 1117 microbiologically documented infections (MDI), 42.07% (n. 470) were caused by Gram-positive bacteria, 34.28% (n. 383) by Gram-negative, 4.83% (n. 54) by fungi, 3.22% (n. 36) by viruses, and in 15.57% (n. 174) of the instances, the pathogen was listed as “bacteria” or “others”. This can be seen in Figure 2.

**Figure 3:**
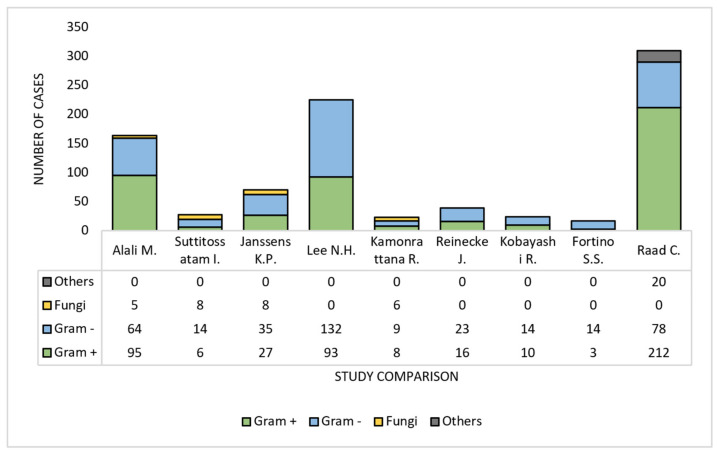
Individual look upon the pathogens involved in the MDIs: Alali M. et al. (2020) [13]; Suttitossatam I. et al. (2020) [15]; Janssens K.P. et al. (2020) [4]; Lee N.H. et al. (2020) [16]; Kamonrattana R. et al. (2019) [19]; Reinecke J. et al. (2018) [22]; Kobayashi R. et al. (2020) [23]; Fortino S.S. et al. (2019) [24]; Raad C. et al. (2021) [25].

When each study was analyzed individually, however, only two of them [12,24] listed Gram-positive bacteria as being the primary pathogen of their MDI, while all the rest described Gram-negative bacteria to be the leading cause of the infections, with Gram-positive pathogens taking up the second place. The third place was occupied by fungi in all but one study [14], where there were more fungi identified as Gram-positive bacteria. This can be seen in Figure 3.

### 3.4. The Empirical Antibiotic of Choice

Concerning the choice of antibiotic administered empirically, there is no consensus regarding which substance to choose, and each hospital follows a protocol best suited for the microbiological profile of infections in that hospital, usually determined through epidemiological reports. Given that the pathogen is unknown, the first-choice antibiotics are the ones with a broad spectrum of antimicrobial effects. 

A great majority of the studies included in this review use third- and fourth-generation cephalosporins in their empirical antibiotic treatment schemes, namely Ceftazidime and Cefepime, and two of the studies [2,19] use Ceftriaxone and Cefoperazone, respectively. This antibiotic class is utilized either alone or in combination with other antibiotics such as aminoglycosides (Amikacin or Gentamicin) and Vancomycin (one study [16] used this antibiotic alone as one of its second options for treating FN next to Meropenem and Teicoplanin). In Haeusler GM et al.’s study [21], one of the analyzed antibiotic combinations was Ceftazidin with Flucloxacillin. 

One-fourth of the studies [21,23,24,25] included here considered Piperacillin/Tazobactam as either their first choice of antibiotic or mentioned this antibiotic combination first in their list of used antibiotic schemes. Six further studies [4,15,18,19,20,25] mentioned using Piperacillin/Tazobactam or Piperacillin alone, but these antibiotics are not necessarily the first line of treatment. Treatment options that include Carbapenem-type antibiotics are schemes in which either Meropenem or Imipenem is utilized alone, without the addition of other antibiotics, and is listed in the studies either as a second option or as one of the antibiotic options, respectively, but it is never the first choice to be mentioned.

## 4. Discussion

Despite the fact that, in most FN cases, there is no MDI, current guidelines [26,27] continue to strongly recommend obtaining blood culture (BC) samples, both from the central venous catheter and periphery, to detect possible bloodstream infections. A study performed in Australia [28] analyzed the differences in yield of pre-and post-antibiotic BC samples. Similar to the results of this review, about 15% of all the FN episodes analyzed have presented with positive BCs. The diagnostic yield of the BC collected before antibiotic administration was significantly greater than after treatment initiation (12.3% compared to 4.4%). Interestingly, the predominant bacteria were Gram-negative bacteria in the positive pre-antibiotic samples, while Gram-positive bacteria dominated positive post-antibiotic samples. 

Regarding the role played by viruses in FN, their detection is limited by the available primers in clinical laboratories. A study performed on a relatively small study population [29] tested the prevalence of viral infections in children with FN with and without signs of acute respiratory infections and compared the data between the two groups. They detected a virus in over three-fourths of the samples collected from the patients presenting with acute respiratory infection signs and in almost half of the samples collected from those without signs, with Rhinovirus and Respiratory Syncytial Virus being the most frequently detected viruses. Although viral infections can coexist with bacterial or fungal infections, and running viral tests might seem, at first glance, to increase the costs of treatment, the authors of this study argue that performing such tests might actually decrease the costs by earlier discontinuation of the antibiotics and by reducing the need of performing certain investigations, thus shortening the hospital stay overall. 

In order to improve the chances of discovering the underlying cause of an FN episode, the same guidelines also recommend obtaining urine samples for urine culture, mentioning that restricting the analysis of this parameter to only symptomatic patients might lead to undiagnosed, or at least underdiagnosed, urinary tract infections. In terms of chest X-rays, neither one of the previously mentioned guidelines recommends performing them unless the patients are presenting with symptoms; the panel of Central American and Caribbean clinicians [27] underlines the fact that pneumonia can be diagnosed clinically and that radiographic results can be nonspecific. 

Concerning the choice of empirically administered antibiotics, monotherapy or combinations with broad-spectrum antibiotics are generally used in treating patients with FN. The guideline for the management of fever and neutropenia in children with cancer and hematopoietic stem-cell transplantation recipients [30] strongly recommends, with a high level of evidence, to start the treatment of high-risk FN episodes with a monotherapy consisting of an antipseudomonal β-lactam, a fourth-generation cephalosporin, or a carbapenem if the patient is hemodynamically stable. Suppose patients are unstable hemodynamically or the center presents a high level of antibiotic resistance. In that case, monotherapy might be deemed insufficient, and an alternative plan of action needs to be elaborated based on the specifics of each clinic. 

On the other hand, the ECIL-8 group [1] recommends using carbapenems with or without other antibiotics solely in clinically unstable patients. For low-risk patients, the formerly mentioned guideline weakly recommends oral administration of antibiotics and, if possible, outpatient setting treatment, mentioning that the readmission rate might increase in the case of orally administered treatment, but when administered parenterally, clinical outcomes between in-patient and out-patient settings proved to be similar. Furthermore, suppose that patient evolution is favorable, or the cause of infection is established; in that case, a step-down or de-escalation approach should be considered by reducing the number of administered antibiotics in cases of combination therapy or starting the patient on a narrower-spectrum antibiotic, one to which the identified bacteria is sensible. De-escalation should be considered after 24–72 h if the patients are clinically stable and have been afebrile for at least 24 h if they are hematologically recovering in the case of high-risk patients and in low-risk patients, even if they are not yet showing signs of hematological recovery [1,27,30].

Regarding the golden hour, which refers to the time interval between patient triage to antibiotic treatment initiation, several guides strongly recommend its use [11,31,32]. The time to antibiotics administration is considered a strong indicator of the quality of care provided in the cancer facility, and any initiative to decrease the mortality and morbidity in patients with FN should promptly address this. These initiatives should include skills training, staff education, up-to-date FN guideline implementation, and feedback on previous performance, as well as tackling more systemic issues for the cancer facilities in low- and middle-income countries, such as logistical issues or understaffed and overworked personnel [7,33].

A few potential limitations to our study can be observed. One possible limitation is that the sample size of selected papers was somewhat small. Another limitation is that the selected studies presented heterogenous data, as some studies focused on the microbiological spectrum, while others focused on antibiotic treatment options. Moreover, some studies were randomized control trials, while others were observational studies (both retrospective and prospective). In order to counter the inherent biases of these types of papers, two researchers were assigned to assess the quality of the selected studies, hence reducing the risk of potential biases, such as selection bias, missing data, or measurement bias. In order to further reduce the risk of bias, the authors encourage a meta-analysis study on the topic.

## 5. Conclusions

Febrile neutropenia is an important and potentially lethal complication that affects mostly children with hematological malignancies. While there seems to be a general consensus on the definition of neutropenia, there are still debates in regard to the temperature point which starts to characterize a febrile episode. Although there are several culture samples being collected at the beginning of or during an FN episode, only about 20% of them come out positive for a pathogen, mostly of a bacterial nature. A viral origin of an FN episode should also be taken into consideration, especially since their current low number seems to be due to limited availability of laboratory primers. As to the main choices of empirical treatment of such episodes, third- and fourth-generation cephalosporins are preferred by most, followed by Piperacillin/Tazobactam, as their first choice of antibiotic. Regarding the outcome of the treatment, in each of the papers reviewed by this study, more than three-fourths of the FN episodes were successfully treated, which positively reflects on the efficiency of the administered medication.

## Figures and Tables

**Table 1:** Studies included in the analysis.

No.	Study Year	Study Type	No. of Studied FN Episodes	No. of Patients	Quality Score
1 [11]	2021	Prospective observational	204	105	11
2 [13]	2020	Retrospective cohort	667	268	10
3 [7]	2020	Randomized multicentric clinical trial	117	69	12
4 [2]	2019	Retrospective cross-sectional	135	135	10
5 [14]	2020	Retrospective cohort	95	95	11
6 [4]	2020	Retrospective cohort	199	118	10
7 [16]	2020	Retrospective cohort	225	164	10
8 [17]	2020	Retrospective cohort	585	265	13
9 [18]	2020	Retrospective and prospective descriptive	563	267	11
10 [19]	2019	Prospective randomized, open-labeled, controlled	118	70	12
11 [20]	2020	Prospective cohort	118	118	9
12 [21]	2020	Prospective, multicentric, non-interventional	858	462	12
13 [22]	2019	Retrospective	194	67	11
14 [23]	2020	Prospective, randomized	394	99	10
15 [24]	2019	Non-blinded randomized controlled clinical trial	100 vs. 76	176	10
16 [25]	2021	Retrospective monocentric descriptive	310	186	11

**Table 2:** Demographic data extracted from the studies.

No.	Male %	Age *	Malignancy	ANC Before Treatment n (Mean)	Central Line n	Last Chemo-Therapy Range (Median)	Fever Days n (Median)
1 [11]	54.91	7.83	Leukemia: 157, Lymphoma: 7, Others: 40	<100: 95 (NR), 100–500: 86 (223)	NR	NR	NR
2 [13]	53.84	10 (median)	ALL 203, AML 115, lymphoma 68, neuroblastoma 110, others 171	667 (NR)	NR	<14 d: 43	1–20 (4)
3 [7]	41.02	7	ALL 56%; Rhabdomyosarcoma 9%; AML 7%	149 (264)	NR	NR	NR
4 [2]	68.88	5.5	ALL: 71, AML: 10, NHL: 10, Blastomas: 17, Sarcomas: 15, Other: 12	135 (120)	NR	<14 d: 88	NR
5 [14]	57.89	6 (median)	ALL: 57, AML 12, Non-leukemia: 26	95 (180)	16	0–147 (8)	0–30 (1)
6 [4]	49.2	8.8	Leukemia: 23, Lymphoma: 7, Sarcoma: 29, Retinoblastoma: 7, Neuroblastoma: 6, Others: 45	199 (NR)	180	NR	NR
7 [16]	50.98	7.75	ALL: 30, AML:41, Lymphoma: 25, Neuroblastoma: 33, Nephroblastomas: 5, Hepatoblastoma: 4, Other: 66	<100: 184 (NR), <500: 194 (NR)	154	1–23 (11)	0–19 (1.2)
8 [17]	NR	11	ALL: 173, AML: 84, Lymphoma: 66, Neuroblastoma: 103, Other: 117	204 (NR)	NR	384	NR
9 [18]	56.55	5.1 (median)	ALL: 114, AML: 44, Lymphoma: 20, Neuroblastoma: 17, Hepatoblastoma: 4, Retinoblastoma: 4, Sarcoma: 16, Other: 48	267 (170)	226	0–148 (10)	4.8 ± 4.7 (mean ± SD)
10 [19]	57.62	7 (median)	ALL: 55, AML:10, Lymphoma: 8, Neuroblastoma: 9, Sarcoma: 8	90 (122)	0	NR	1–4 (2)
11 [20]	66.94	4.7 (median)	Hematological: 82, Solid tumors: 36	118 (110)	NR	<7 d: 85, >7 d: 33	4–6 (5)
12 [21]	51.63	5.8 (median)	ALL: 375, AML: 67, NHL: 55, Hodgkin: 11, Neuroblastoma: 48, Medulloblastoma: 37, Nephroblastoma: 19, Sarcoma: 177, Other: 88	858 (NR)	845	NR	NR
13 [22]	47.76	6.7	ALL: 44, AML: 23	67 (NR)	184	NR	NR
14 [23]	63.63	9.9 (median)	ALL: 52, AML: 12, NHL:9, Solid tumors: 14, Other: 11	394 (103)	380	NR	NR
15 [24]	59.55	9.5 (median)	ALL: 24, AML: 10, NHL: 40, Solid tumors: 76, Other: 26)	176 (NR)	NR	NR	NR
16 [25]	NR	5.3	ALL: 79, AML: 21, NHL:11, Solid tumors: 114, Others: 37	NR	310	NR	NR

* Data reported as mean, unless specified differently; ALL, acute lymphocytic leukemia; AML, acute myeloid leukemia; NHL, non-Hodgkin lymphoma; NR, not reported.

**Table 3:** Etiologic agents’ data extracted from the studies.

No.	Infection Site	Hospital Stay/Duration of Antibiotic Therapy	Etiological Agent	MDR Strains	Most Frequent Bacterium
1 [11]	Chest: 40, BSI: 14, GI: 29, Others: 36, Unknown: 69	13 ± 8.5	Bacterial: 27 (64.5% G-), Viral: 7, Fungal: 3	NR	Klebsiella pneumoniae
2 [13]	BSI: 143	NR	G+ 95, G- 64, Other Bacteria 17, Fungal: 5	21	Alpha-hemolytic streptococcus: 35
3 [7]	NR	4.25 ± 2.5	NR	NR	NR
4 [2]	Chest: 15, GI: 5, Urinary: 2, Unknown: 113	NR	NR	NR	NR
5 [14]	BSI: 12, Urinary: 8, GI: 2, Respiratory: 8, Other: 2	NR	G- bacteria: 14, G+ bacteria: 5, Viral: 2, Fungal: 5	2	Klebsiella pneumoniae
6 [4]	NR	NR	G- bacteria: 35, G+ bacteria: 27, Fungal: 8	NR	Klebsiella pneumoniae (14.2%) and Pseudomonas aeruginosa (14.2%
7 [16]	BSI: 221, Skin/soft tissue: 14, GI: 30, Oral: 16, Other: 5	5 to 93	G- bacteria: 132, G+ bacteria: 93	60	Escherichia coli: 98
8 [17]	BSI: 141	10 (median)	G- bacteria: 64, G+ bacteria: 199, Fungal: 47	NR	NR
9 [18]	NR	NR	Bacterial: 154, Viral: 32, Fungal: 27	NR	NR
10 [19]	BSI: 6, Urinary: 11	7 to 12	G- bacteria: 13, G+ bacteria: 4	NR	Escherichia coli: 3
11 [20]	Respiratory: 81, GI: 10, Urinary: 1, Unknown: 26	5 to 11	NR	NR	NR
12 [18]	NR	NR	NR	NR	NR
13 [22]	BSI: 67	2 to 24	G- bacteria: 23, G+ bacteria: 16	5	Escherichia coli: 10
14 [23]	NR	2 to 19	G- bacteria: 16, G+ bacteria: 8	4	Staphylococcus. aureus: 4
15 [24]	NR	NR	G- bacteria: 14	2	Pseudomonas aeruginosa: 5
16 [25]	BSI: 310	10 to 21	G- bacteria: 25.2%, G+ bacteria: 68.4%,	65	Coagulase-Negative Staphylococci: 34

BSI: bloodstream infections; NR: not reported; NA: not applicable; G-: Gram negative; G+: Gram positive.

**Table 4:** Treatment data extracted from the studies.

No.	Most Used Antibiotic	Second Most Used Antibiotic	Time to Antibiotic Administration (min)	Treatment Modifications *N*	Other Anti-Infectious Medication
1 [11]	Cefepime	Meropenem + Vancomycin	47.17	NR	NR
2 [13]	Ceftazidime: 50%	Ceftazidime + Vancomycin: 33%	NR	NR	Antifungals
3 [7]	Cefepime	Cefixime	NR	3	NA
4 [2]	Ceftriaxone + Gentamycin: 97	Ceftriaxone: 6	NR	22	Antiviral: 30, Antifungal: 52
5 [14]	Ceftazidime + Amikacin	Piperacillin/Tazobactam	<120	NR	NR
6 [4]	Cefepime	Meropenem or Piperacillin/Tazobactam	NR	NR	NR
7 [16]	Cefepime: 121	Cefepime + Amikacin: 83	120 (median)	57	NR
8 [17]	Ceftazidime + Vancomycin	Cefepime + Vancomycin	NR	NR	Antifungals
9 [18]	NR	NR	NR	NR	Antifungal: 123
10 [19]	Piperacillin/Tazobactam: 59	Ceftazidime + Amikacin: 59	30	44	Antifungal: 21
11 [20]	Amoxicillin/Clavulanate + Amikacin (respiratory), Cefoperazone/Sulbactam + Metronidazole (GI)	Meropenem + Vancomycin or Teicoplanin	NR	34	Antifungal: 19
12 [21]	NR	NR	552	NR	Antiviral: 72, Antifungal: 290
13 [22]	Cefepime: 157	Cefepime + Vancomycin: 16	NR	35	NA
14 [23]	Meropenem: 200	Piperacillin/Tazobactam: 193	NR	NA	NA
15 [24]	Intermittent Piperacillin/Tazobactam: 100	Continuous Piperacillin/Tazobactam: 76	NR	6	NA
16 [25]	Vancomycin: 134	Amikacin: 90	NR	NR	Antifungal: 44

**Table 5:** Main outcomes as reported from the studied records.

No.	Persistent Fever	Central Line Removal	Sepsis	ICU	Deaths	Treated
1 [11]	NR	NR	32	23	4	200
2 [13]	NR	NR	NR	4	35	139
3 [7]	1	NR	NR	NR	0	117
4 [2]	2	NR	NR	NR	7	95
5 [14]	NR	NR	3	11	0	95
6 [4]	NR	NR	NR	8	1	117
7 [16]	NR	1	16	4	0	225
8 [17]	NR	NR	NR	87	8	577
9 [18]	NR	NR	NR	78	21	542
10 [19]	NR	NR	NA	NR	0	118
11 [20]	11	NR	5	8	6	112
12 [21]	NR	NR	13	24	4	424
13 [22]	NR	1	1	5	1	66
14 [23]	NR	NR	NR	NR	0	288
15 [24]	1	NR	1	NR	2	136
16 [25]	NR	60	14	0	0	310

## Data Availability

Data are available upon request.
